# Fenofibrate Induces Ketone Body Production in Melanoma and Glioblastoma Cells

**DOI:** 10.3389/fendo.2016.00005

**Published:** 2016-02-02

**Authors:** Maja M. Grabacka, Anna Wilk, Anna Antonczyk, Paula Banks, Emilia Walczyk-Tytko, Matthew Dean, Malgorzata Pierzchalska, Krzysztof Reiss

**Affiliations:** ^1^Department of Food Biotechnology, Faculty of Food Technology, University of Agriculture, Krakow, Poland; ^2^Molecular and Metabolic Oncology Program, Department of Oncologic Sciences, Mitchell Cancer Institute, University of South Alabama, Mobile, AL, USA; ^3^Department of Human Nutrition, Faculty of Food Technology, University of Agriculture, Krakow, Poland; ^4^Neurological Cancer Research, Stanley S Scott Cancer Center, Louisiana State University Health Sciences Center, New Orleans, LA, USA

**Keywords:** peroxisome proliferator-activated receptor alpha, beta-hydroxybutyrate, 3-hydroxy-3-methylglutaryl-CoA synthetase 2, transketolase

## Abstract

Ketone bodies [beta-hydroxybutyrate (bHB) and acetoacetate] are mainly produced in the liver during prolonged fasting or starvation. bHB is a very efficient energy substrate for sustaining ATP production in peripheral tissues; importantly, its consumption is preferred over glucose. However, the majority of malignant cells, particularly cancer cells of neuroectodermal origin such as glioblastoma, are not able to use ketone bodies as a source of energy. Here, we report a novel observation that fenofibrate, a synthetic peroxisome proliferator-activated receptor alpha (PPARa) agonist, induces bHB production in melanoma and glioblastoma cells, as well as in neurospheres composed of non-transformed cells. Unexpectedly, this effect is not dependent on PPARa activity or its expression level. The fenofibrate-induced ketogenesis is accompanied by growth arrest and downregulation of transketolase, but the NADP/NADPH and GSH/GSSG ratios remain unaffected. Our results reveal a new, intriguing aspect of cancer cell biology and highlight the benefits of fenofibrate as a supplement to both canonical and dietary (ketogenic) therapeutic approaches against glioblastoma.

## Introduction

Adaptation to limited nutritional resources due to a constantly changing environment is the most elementary challenge for organisms that are subjected to the pressures of natural selection. Metabolic flexibility that enables fitness and survival in cases of food shortage constitutes the basis of evolutionary success in terms of reproduction and preservation of offspring. Creativity in the substitution of glucose as a source of carbon and energy with other substrates has been the main driving force in prokaryotic evolution, as well as a necessity in the course of the development of multicellular organisms. Warm-blooded animals, such as mammals, which have a constantly highly active metabolism, are particularly prone to the detrimental effects of prolonged food shortages. Development of highly complex structures like the human brain, which consumes about 20% of the body’s entire energy resources, complicated the matter even further. Therefore, tightly regulated metabolic processes such as ketone body generation evolved as a rescue program during starvation and provided a significant survival advantage in environments where resources were short.

Ketone bodies, namely acetoacetate, acetone, and beta-hydroxybutyrate (bHB) are produced in the liver during an extensive fatty acid oxidation. They are the products of acetoacetyl-CoA and acetyl-CoA condensation. This reaction, a rate-limiting step of ketogenesis, leads to the formation of 3-hydroxy-3-methylglutaryl-CoA (HMG-CoA) and is catalyzed by mitochondrial HMG-CoA synthetase (HMGCS2). Acetoacetate and bHB serve as highly efficient metabolic substrates and are utilized by peripheral tissues for energy production, at an even higher rate than glucose. Ketogenesis is regulated by several proteins that act as energy sensors, such as peroxisome proliferator-activated receptor alpha (PPARa), peroxisome proliferator-activated receptor gamma coactivator 1 alpha (PGC-1a), sirtuins, and AMP-dependent protein kinase (AMPK) (1–4).

Peroxisome proliferator-activated receptor alpha is a nuclear receptor that acts as the key transcription factor responsible for the mobilization of lipid stores, fatty acid oxidation, and ketogenesis during fasting (5, 6). PPARa is activated by free fatty acids and their derivatives (particularly CoA-SH esters and amides), which are released during fat mobilization; therefore, it acts as a metabolic switch. Pharmacological agonists of PPARa, especially the hypolipidemic drug fenofibrate, are also able to induce fatty acid oxidation and ketogenesis in hepatocytes (7–9). PPARa is directly responsible for driving the transcription of HMGCS2 during fasting or in response to a ketogenic diet (KD). Additionally, there is a functional peroxisome proliferator response element (PPRE) in the HMGCS2 promoter (−105 to −92) (10). Further regulation of HMGCS2 expression is unique, as it involves the binding of the HMGCS2 protein itself to the PPARa receptor. The resulting complex is then translocated into the nucleus, where it triggers HMGCS2 transcription (11). Further, HMGCS2 activity is also upregulated by post-translational modifications like deacetylation and desuccinylation on multiple lysine residues by the mitochondrial sirtuins Sirt3 and Sirt5, respectively, and palmitoilation of cysteine in the active site (12–14). Interestingly, HMGCS1, a cytosolic counterpart of HMGCS2 involved in steroid synthesis, is neither transcriptionally regulated by PPARa nor does it physically interact with this receptor (15, 16).

Principally hepatocytes and to a lesser extent epithelia from the kidneys and intestines are responsible for ketogenesis under physiological conditions, as these tissues possess the proper enzymatic machinery. Ketogenesis is switched on during prolonged fasting (over 1 day), when the glucose level in the blood is very low and higher levels of glucagon stimulate lipolysis and fatty acid flux from the adipose tissue. Glycogen from the liver and skeletal muscles is sufficient to sustain blood glucose levels for approximately 24 h, but after glycogen stocks are used up, reserves of fat are mobilized and free fatty acids released (17–19). Fatty acids are taken up by the liver and peripheral tissues and metabolized by beta-oxidation to acetyl-CoA, which is then oxidized completely in the TCA cycle. Lipid catabolism is carried out at a high rate, especially in the liver, where accumulated acetyl-CoA is transformed to beta-HB and released into the blood. All extrahepatic tissues including cardiac and skeletal muscles absorb and oxidize ketone bodies readily. The hierarchy of energy metabolite consumption causes ketone bodies to be used instead of glucose, which is spared for the brain (19). Utilization of ketone bodies significantly reduces the need for gluconeogenesis, the substrates for which are mostly provided by catabolism of protein-derived aminoacids (this process causes slow muscle wasting) or glycerol through the process of triglyceride de-esterification.

Interestingly, in contrast to other peripheral tissues, the brain is very limited in its capacity to utilize free fatty acids. There are a few reasons for this: (i) fatty acid oxidation generates large amounts of reduced flavin adenine dinucleotide (FADH_2_) that leads to enhanced generation of ROS by electron transport flavoprotein-ubiquinone oxidoreductase, which causes membrane peroxidation; (ii) fatty acid transport to the brain and oxidation within the brain is far too slow to provide enough ATP during periods of rapid neuronal synaptic activity and impulse generation; (iii) oxygen concentration in brain tissue is low, whereas the amount of oxygen needed for complete oxidation of long chain fatty acids is high – therefore complete oxidation could increase the risk of hypoxia in adjacent regions (20). Metabolism of ketone bodies eliminates all these drawbacks and provides a high-energy yield – roughly 23–26 ATP/one bHB molecule. Moreover, bHB is the most efficient fuel per molecule of oxygen consumed when compared to glucose, pyruvate, or free fatty acids, due to its ability to widen the redox potential gap between the respiratory complex I (NADH/NAD) and CoQ/CoQH_2_ (21–24).

Utilization of ketone bodies as a source of energy requires expression of enzymes involved in acetoacetate and bHB catabolism, such as succinyl-CoA:acetoacetate-CoA transferase (SCOT), also known as 3-oxoacid-CoA transferase (OXCT1) (EC 2.8.3.5); 3-oxoacyl-CoA thiolase, also known as acetyl-CoA actyltransferase (ACAT) (EC 2.3.1.9); and bHB dehydrogenase (EC 1.1.1.30). All of these enzymes are present in peripheral tissues at various levels; however, SCOT is absent in the liver to prevent a futile cycle of bHB synthesis and utilization (25).

In general, oncogenic transformation is associated with increased glucose consumption – a high rate of glycolysis, even in normoxia (the so-called Warburg effect), as well as avid consumption and metabolism of glutamine (glutaminolysis) (26, 27). Neoplastic cells, however, are not capable of ketone body consumption because the majority of cancers do not express the required enzymatic machinery. This phenomenon has been particularly well studied in brain tumor models. Malignant cells derived from brain tissue (neuroblastomas, glioblastomas multiforme, astrocytomas, and schwannomas) either do not express SCOT and ACAT or express these enzymes at very low levels, making them incapable of using ketone body oxidation for ATP production (25, 28). In fact, neuroblastoma and glioblastoma cells are only able to utilize ketone bodies as substrates for lipid synthesis (29–31). Cancer cells that are not able to metabolize bHB and acetoacetate frequently suffer from ketone body-induced toxicity, which has already been reported in neuroblastoma (30). Moreover, ketone bodies exert strong anti-proliferative and pro-apoptotic effects in melanoma, pancreatic, gastric, colon, and cervical cancer cells, as well as in transformed lymphoblasts (32–35). These observations provide a rationale for proposing a dietary restricted KD as a supportive therapy against malignant glioma. Calorie/dietary restriction combined with a high fat/low carbohydrate (ketogenic) diet decreases blood glucose levels and simultaneously elevates bHB levels (36). This type of dietary intervention has already been shown to have very encouraging effects, namely inhibition of both tumor growth and neovascularization, as well as improved overall survival in patients and in various *in vivo* models of brain cancers (37–41). The Scheck team was able to achieve remarkable results, when they applied radiation therapy along with the KD, they were able to achieve complete remission of malignant glioma (42). In addition, the ability to synthesize ketone bodies is frequently lost during oncogenic transformation of colonic epithelia, where the c-Myc oncogene transcriptionally repressed transcription of the HMGCS2 gene in 90% of colon carcinoma samples tested (43).

All these lines of evidence strongly suggest that ketogenesis is a process that is observed only under very particular physiological circumstances, which are absent in cancer cells. Furthermore, cancer cells neither prefer this kind of energy substrate nor flourish in a ketogenic environment, which does not suit the needs of highly proliferating cells. However, in this paper, we present a novel observation that malignant cells of neuroectodermal origin, namely melanoma and glioblastoma cells, are capable of efficient synthesis and release of bHB when treated with a synthetic PPARa agonist, fenofibrate. Unexpectedly, the induced ketogenesis seems to be independent of PPARa expression level or its activity in these cells.

## Experimental Procedures

### Cell Culture and Treatment

Murine melanoma B16 F10 (ATCC# CRL-6475) cells were cultured in RPMI 1640 medium (Pan-Biotech, Germany) supplemented with 10% fetal bovine serum (FBS, Pan-Biotech, Germany), mixture of antibiotics and antimycotics (penicillin 50 U/ml, streptomycin 50 μg/ml, and amphotericin B 250 ng/ml), and 2 mM glutamine (Sigma Aldrich, Germany). Human glioblastoma cell line LN-229 (ATCC# CRL-2611) cultures were maintained in DMEM (Gibco, Thermo Fischer, USA) supplemented with antibiotics, 10% FBS, and 2 mM glutamine. Both cell lines were kept at 37°C and 5% CO_2_ atmosphere. Primary neurospheres cultures were obtained by isolating neural progenitor cells from whole-brains of embryonic day 17 C57BL/6 mice according to the previously published protocol (44). Isolated progenitor cells were cultured in poly-2-hydroxyethyl methacrylate (Sigma Aldrich, USA)-coated dishes in Neurobasal media supplemented with B27, Glutamax, N2, bFGF (Gibco, Thermo Fischer, USA), heparin (Stem Cell Technologies, USA), and 20 ng/ml EGF (Sigma Aldrich, USA). Neursopheres were passaged and plated on poly-d-lysine/laminin (Sigma Aldrich, USA)-coated glass chamber slides. Following 4 days of differentiation, cells were treated with a vehicle (DMSO, Sigma Aldrich, USA) or fenofibrate (FF, Sigma Aldrich, USA) for 48 h, and media was collected for β-HB assay. FF was added to the fresh cell culture media at final concentration of 50 μM (Sigma Aldrich, USA, diluted from DMSO stock), and the PPARa inhibitor MK886 (Merck Millipore, USA) was used at 10 μM (45), whereas the final concentration of vehicle when added to the control cultures did not exceed 0.05% *v*/*v*.

### Plasmids and Transfections

In order to knock down the PPARa protein level, a commercially available plasmid driving shRNA against PPARa was used (Santa Cruz Biotechnology, sc-36307-sh, USA), along with a plasmid producing irrelevant, control shRNA molecules (Control shRNA Plasmid A, Santa Cruz Biotechnology, sc-108060, USA) to generate a reference cell line. The B16 F10 cells were plated on the 60-mm dishes (200,000 cells per dish) transiently transfected with 1.75 μg of plasmids using Lipofectamine 2000 (Thermo Fisher, USA) reagent, and for the stable clones, the selection with puromycin (10 μg/ml) was applied, starting 72 h after the transfection. The cell lines with PPARa overexpression was prepared by the transient or stable transfections with pcDNA6.2/N-EmGFP-DEST vector (Thermo Fisher, USA) with inserted human PPARa ORF under the control of CMV promoter. The expression vector was prepared using Gateway^®^ Cloning System (Thermo Fisher, USA). Briefly, PPARa ORF sequence (CR456547_1) was synthesized, optimized for the expression in human cells, and cloned into the pDONR221 Entry Vector (GeneArt, Thermo Fisher, USA). Subsequently, the ORF insert was transferred into the pcDNA6.2/N-EmGFP-DEST Destination Vector via Clonase II Recombination Reaction. The stable clones were selected with blasticidin-S (20 μg/ml), starting 72 h after the transfection. Transfection efficiency (in the cells co-transfected with pDsRed2-Mito plasmid, Clontech laboratories, USA) and the expression of PPARa-EmGFP fusion protein were monitored in living cells or in fixed slides counterstained with DAPI in the fluorescent confocal microscope (Olympus Fluoview FV1000 laser scanning biological microscope).

### Beta-Hydroxybutyrate Assay

The cells seeded in 12-well plates were treated with DMSO (control) or fenofibrate and/or MK886 for 48 h. The media were collected and centrifuged to eliminate any possible cell debris and then processed immediately or frozen in −80°C. The concentration of bHB in the cell culture media aliquots of 50 μL was measured using bHB colorimetric assay kit (Biovision #K632, USA), according to the manufacturer’s instructions. The bHB concentrations were calculated from the 450 nm absorbance recorded from the microplate reader and the standard curve prepared with the 1 mM bHB solution included in the kit. Every time the bHB production was normalized to the total cell number in each well. For this purpose and for assessment of cell proliferation, cells were fixed and stained with crystal violet solution, and the cell numbers were determined by a spectrophotometric method, according to Kueng et al. (46).

### Immunoblotting

Total protein extracts were isolated from LN-229 and B16 F10 cells using cell lysis buffer from Cell Signaling Technology, USA (#9803) supplemented with 1 mM PMSF, protease, and phosphatase inhibitor cocktails (Roche Applied Science Complete^®^ and PhosphoStop^®^). Sample preparation and immunoblotting were performed according to the standard procedures described in our previous studies (47). The following primary antibodies were used: anti-PPARa, rabbit polyclonal SAB2104354 (1:2,000 dilution) Sigma Aldrich, USA; anti-PGC-1a; anti-HMGCS1; anti-HMGCS2 rabbit polyclonal from Santa Cruz Biotechnology (all in 1:1,000 dilution), USA; anti-phospho- and total ATP-citrate lyase (ACL); anti-Acetyl-CoA synthetase (AceS1); anti-pyruvate kinase M2 (PKM2); and anti-transketolase (TKT) rabbit monoclonal from Cell Signaling Technology (USA, all in 1:1,000 dilution). For loading control, anti-Grb2 mouse monoclonal antibody (1:3,000, BD Bioscience, San Jose, CA, USA) was used. For the signal detection anti-rabbit IgG HRP-linked antibodies (#7074 Cell Signaling Technology, USA), anti-mouse IgG (H + L), HRP conjugated antibodies (Thermo Fisher, USA), and a chemiluminescent peroxidase substrate SignalFire (Cell Signaling Technology, USA) were used. Densitometric analysis of immunoblots was performed using ImageJ software (NIH, USA).

### Mitochondrial Oxygen Consumption

The mitochondrial oxygen consumption rates in the immediate response to fenofibrate were evaluated in the B16 F10 Ctrl shRNA or PPARa shRNA cells using the Extracellular Flux Analyzer XF24 (Seahorse Biosciences, North Billerica, MA, USA), as previously described (48). Briefly, the day before each assay, the cells were plated in 24-well plates (Seahorse Biosciences, USA) in growth-supporting medium (25,000 cells/well). At the time of measurement, growth media were replaced with serum-free XF assay medium (Seahorse Biosciences, USA), and cartridges equipped with oxygen-sensitive and pH-sensitive probes (Seahorse Biosciences, USA) were placed above the cells. The oxygen consumption rate (OCR; indicative of mitochondrial respiration) and extracellular acidification rate (ECAR; indicative of glycolysis) were evaluated after injecting FF (50 μM) or vehicle (DMSO), followed by injections of metabolic toxins: oligomycin (inhibitor of ATP synthase; complex V of the Electron Transport Chain, ETC; 0.5 μM), carbonyl cyanide-*p*-trifluoromethoxyphenylhydrazone (FCCP; uncoupling factor; 0.75 μM), and rotenone (inhibitor of mitochondrial complex I of ETC; 0.75 μM).

### Cell Cycle and Clonogenic Assays

Cell cycle distribution was assessed by flow cytometry (GUAVA EasyCyte8HT, Merck Millipore, USA). Briefly, the aliquots of 1 × 10^6^ cells/ml were fixed in 70% ethanol at −20°C overnight. Next, cells were centrifuged at 1,600 rpm for 5 min, and the resulting pellets resuspended in Guava Cell Cycle reagent (Merck Millipore, USA). Cell cycle was evaluated using specialized software CellCycle included in GuavaSoft 1.1.

LN-229 and wt B16 F10, PPARa shRNA, Ctrl shRNA, or PPARa-GFP cells were plated at the clonal density (1,000 cells per well in a 6-well plate). After 24 h, cells were treated with FF and/or MK-886. Fresh media with tested compounds were given every second day for the course of 12 days. Control cells were treated with DMSO. At the end of each experiment, cells were fixed and stained with the 0.25% crystal violet solution in methanol, air dried, and the colonies were counted. All the conditions were tested in duplicates or triplicates, and the experiments were performed at least three times.

### NADP/NADPH and GSH/GSSG Assays

For the calculation of the NADP/NADPH and GSH/GSSG ratios, cells were plated in 24-well plates (20,000/well) and treated with DMSO (control) or FF for 48 h. Next, cells were lysed, followed by the luminometric assays for NADP, NADPH, GSH and total GSH, and GSSG using the NADP/NADPH-Glo and GSH/GSSG-Glo kits (Promega, Germany), according to the attached instructions. The luminescence signal was recorded with the tube GloMax 20/20 luminometer (Promega, Germany).

### Statistical Analysis

Each experimental data points were done in triplicate, and the independent experiments were repeated two or three times. The data were analyzed using Statistica 10 StatSoft (USA) software, performing one-way or two-way ANOVA and *post hoc* Tukey tests; differences between the control and experimental groups were considered significant for *P* values lower than 0.05.

## Results

### Fenofibrate Triggers bHB Production Regardless of PPARa Expression or Activity Status

**Figure 1:**
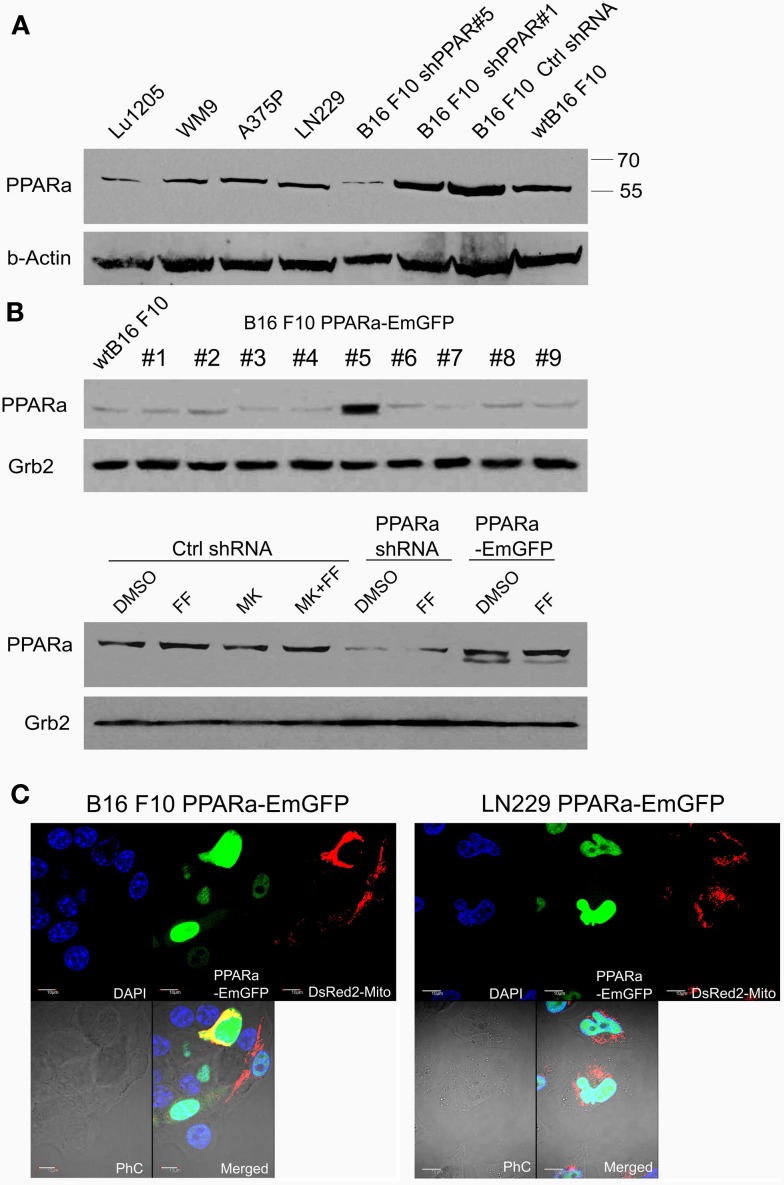
**Detection of the PPARa protein (A) in various human melanoma cell lines (Lu1205, WM9, A375P), human glioblastoma (LN229), and various clones of murine melanoma B16 F10 sublines expressing PPARa shRNA (shPPAR#1 and shPPAR#5, shown as examples) or control shRNA (Ctrl#2)**. Wt B16 F10 whole cell extract served as a reference. In the shPPAR#1 clone, the silencing was not successful, and for further experiments, B16 shPPAR#5 subline showing about 85% reduction of PPARa expression was chosen. The B16 F10 stable cell line overexpressing PPARa-EmGFP fusion protein (#5) was selected **(B)**, wt B16 F10 whole cell extract is used for comparison of PPARa levels. Fenofibrate (FF, 50 μM, 48 h) and MK886 (10 μM, 48 h) do not affect PPARa protein level; DMSO was used as a control **(B)**. The expression and subcellular (nuclear) localization of the fusion protein PPARa-EmGFP (green) is shown in B16 F10 and LN229 cells **(C)**. Transfection with pDsRed2-Mito plasmid (red) indicated the transfection efficiency; nuclei are counterstained with DAPI (blue). Scale bar – 10 μm.

Although fenofibrate is a well-known PPARa agonist used clinically to normalize blood lipoprotein profiles, many of its recently described anticancer activities do not involve the PPARa-driven transactivation mechanism. Our previous *in vitro* and *in vivo* studies conducted on various malignant cell lines of neurocetodermal origin demonstrated that fenofibrate inhibited proliferation, migration, invasion, metastatic tumor formation, and affected energy homeostasis, which led to metabolic catastrophe (48, 49). The latter effects seem to be promising and might aid the dietary ketogenic regimens currently being developed to support the conventional chemotherapies and radiotherapies used against gliomas. Therefore, in order to further investigate the strong impact that fenofibrate has on the cellular metabolism and to distinguish the receptor dependent and independent effects, we performed the experiments on neoplastic cell lines with varying levels of PPARa expression. The cell lines used were aggressive B16 F10 murine melanoma and human glioblastoma LN229, which both show detectable levels of endogenous PPARa expression (Figure 1A). PPARa knock down in B16 F10 cells was achieved through transcriptional silencing with specific shRNA, which resulted in an 85% decrease in its protein level, as estimated by densitometric analysis (not shown). The B16 F10 clone that stably overexpressed the PPARa-EmGFP fusion protein showed an over 12-fold increase in the level of receptor expression when compared to wt B16 F10 (Figure 1B). In the case of LN229 cells, transient transfection with a PPARa-EmGFP expression vector resulted in an enrichment of the population of cells which expressed high levels of nuclear PPARa-EmGFP (Figure 1C).

**Figure 2:**
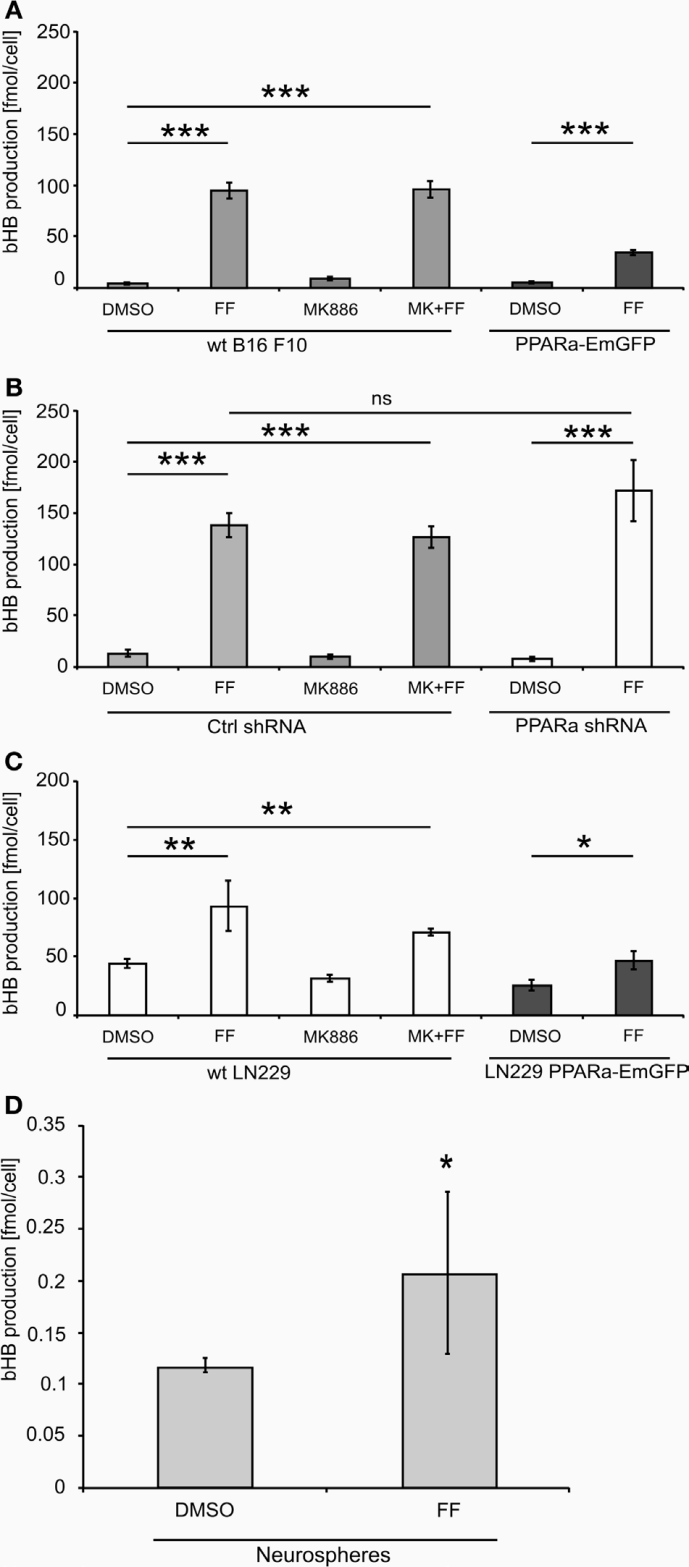
**Beta-hydroxybutyrate (bHB) production in melanoma (A,B), glioblastoma cells (C) varying in the PPARa level and in the neurosphere culture (D)**. FF – fenofibrate, MK886 – the PPARa inhibitor MK886, cell line names: PPARa shRNA – PPARa downregulation, PPARa-EmGFP – PPARa overexpression. Statistical significance of the observed differences are shown as asterisks: *P < 0.05, **P < 0.001, ***P < 0.0001.

Treatment with fenofibrate (50 μM) induced ketogenesis in both B16 F10 and LN229 cells, which manifested in the production and release of bHB into the cell culture medium (Figure 2).The amount of bHB in the medium of FF-treated samples was 24-fold higher compared to DMSO-treated B16 F10 cells. Incubation with the synthetic PPARa inhibitor MK886 did not reverse this effect (Figure 2A). Similarly, the cells treated with shRNA against PPARa and control cells showed the same trend as wt B16 F10, whereby fenofibrate efficiently induced ketogenesis, which resulted in a 24-fold increase in bHB production compared to DMSO-treated cells (Figure 2B). In B16 F10 cells transiently overexpressing PPARa, fenofibrate was also able to trigger ketogenesis (Figure 2A). LN229 glioblastoma cells responded to fenofibrate similarly to the melanoma cells, with enhanced bHB production, regardless of the presence of the PPARa inhibitor MK886 or the level of PPARa expression (Figure 2C). Noteworthy was that the basal level of bHB production in DMSO-treated LN229 cells was about three to four times higher than in B16 F10 cells. This may be explained by the retained intrinsic capability of ketogenesis observed in normal astrocytes, which is absent in melanocytes.

Intrigued by these observations, we tested whether fenofibrate had a similar effect on normal cells. We used neurospheres, which are multicellular organoids of neurons and glia and serve as a simplified model of the normal brain tissue. Indeed, fenofibrate treatment (50 μM, 48 h) induced a statistically significant increase in bHB production (Figure 2D), albeit the differences between control and fenofibrate stimulated cultures were less pronounced than in case of malignant cells. Due to the difficulty in assessing the cell number precisely, the bHB concentrations measured in neurosphere culture samples were normalized to the amount of protein. Thus, in order to compare the values obtained for neurospheres with those from melanoma and glioblastoma cells, we performed a recalculation. Assuming that one cell contains approximately 240 pg of protein [in mammalian cells proteins account for 60% dry mass and average dry mass of a mouse embryonic fibroblast is 400 pg (50)], we can estimate the mean bHB production by control wt B16 F10 cells as 2.83 nmol/(μL*mg of protein) and 18.28 nmol/(μL*mg of protein) in the fenofibrate-treated group; in LN229 glioblastoma cells, it was 3.66 and 7.82 nmol/(μL*mg of protein) for the control and fenofibrate groups, respectively. These results are considerably higher than the mean values for neurospheres that yielded 0.117 and 0.207 nmol/(μL*mg of protein) in control and fenofibrate-treated groups, respectively. This may be explained by the fact that astrocytes, which are capable of ketogenesis, make up only a fraction of the total cell population in the neurosphere. Nevertheless, it seems that the pro-ketogenic activity of fenofibrate is not restricted to malignant cells; rather, it appears that healthy cells can also respond similarly.

### Ketogenesis in B16 F10 Cells Is Accompanied by a Time-Dependent Increase in the HMGCS2 Protein Level

**Figure 3:**
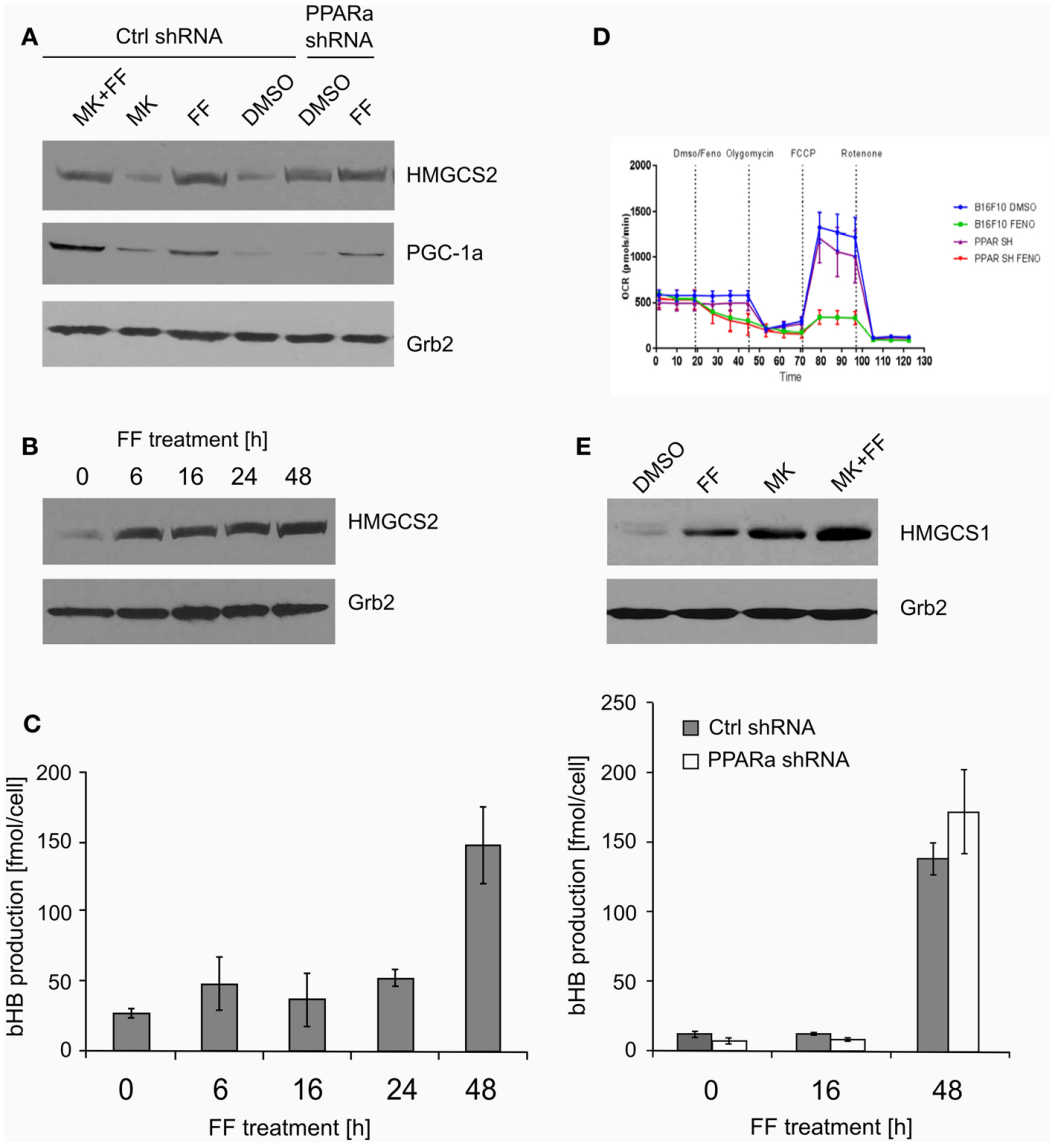
**The expression of the key ketogenic enzyme mitochondrial HMGCS2 and PGC-1a factor responsible for the mitochondrial biogenesis (A) in the cells treated with fenofibrate (FF) and the PPARa inhibitor (MK886) in the B16 F10 cells expressing normal (Ctrl shRNA) or reduced (PPARa shRNA) levels**. Fenofibrate induces a time-dependent increase of the HMGCS2 expression **(B)**, which precedes a significant increase in the beta-hydroxybutyrate (bHB) production **(C)**, independently of the PPARa level. Fenofibrate represses the oxygen consumption **(D)** in both normal and knocked down PPARa cells (Ctrl shRNA and PPARa shRNA, respectively). Fenofibrate and MK886 increase HMGCS1 expression in an additive fashion in the B16 F10 cells **(E)**.

Both ketogenesis and fatty acid beta-oxidation take place in the mitochondria; thus, properly functioning mitochondria are essential for these processes. Mitochondrial HMGCS2 is the rate-limiting enzyme of the ketogenesis pathway (14), so we checked if its level increases after fenofibrate treatment. Indeed, HMGCS2 was upregulated in the cells treated with fenofibrate, regardless of the presence of MK886. Surprisingly, the basal HMGCS2 level was even higher in the B16 F10 cells with reduced PPARa expression (Figure 3A). This suggests that in the melanoma cells we studied, either PPARa does not play a role as an HMGCS2 transactivator or that the process might also be regulated downstream of transcription – perhaps through altered protein stability or turnover rate. Nevertheless, this observation drew our attention, and so we decided to examine both the ketogenic response of B16 F10 cells to fenofibrate and the levels of HMGCS2 following various periods of incubation with fenofibrate. As shown in Figure 3B, although the increase in HMGCS2 synthesis was triggered after only a 6-h incubation with fenofibrate, a significant accumulation of bHB in the cell culture medium was not detected until 48 h after the start of the treatment (Figure 3C). The time course of bHB production was similar in both B16 F10 ctrl shRNA and PPARa shRNA generating cells (Figure 3C), which suggests that although HMGCS2 expression can be triggered quite rapidly, metabolic adaptation to the fenofibrate challenge takes nearly 2 days.

The increased HMGCS2 expression induced by fenofibrate was accompanied by the upregulation of PGC-1a levels (Figure 3A), which may be reflected in the stimulated mitochondrial biogenesis and intensification of mitochondrial metabolism. This could also serve as a compensatory mechanism, since our previous experiments showed that in LN229 cells, fenofibrate penetrated the mitochondria and acutely – albeit reversibly – repressed oxygen consumption through inhibition of the respiratory complex I, in a manner similar to rotenone (48). Indeed, similar observations were made for B16 F10 cells expressing normal or reduced PPARa levels (Figure 3D). Within minutes of fenofibrate addition to the monolayer culture, the oxygen consumption rate dropped significantly. Next, exposure to several metabolic toxins, such as oligomycin (complex V – ATP synthase inhibitor), FCCP (uncoupler that depolarizes mitochondrial inner membrane), and rotenone (irreversible complex I inhibitor) was used in order to obtain a respiratory profile of the cells following fenofibrate treatment. We demonstrated that fenofibrate-treated cells lost the ability to respond to oligomycin (as if ATP synthesis was already very weak) and barely responded to FCCP due to the lack of a proton gradient in the mitochondria (Figure 3D). Noteworthy, the reduction of PPARa levels did not significantly change the respiratory profiles of B16 F10 cells in either vehicle or fenofibrate treated cells.

HMGCS1 – the cytoplasmic counterpart of mitochondrial HMGCS2 – is involved in cholesterol biosynthesis but not ketogenesis. Transcription of HMGCS1 is not normally regulated by PPARa; thus, the observed positive effect of both fenofibrate and MK886 on HMGCS1 protein level was quite unexpected, particularly the additive stimulation of HMGCS1 expression in the cells treated with both fenofibrate and MK886 in wt B16 F10 (Figure 3E).

### Fenofibrate Treatment Affects Glucose Catabolism and Lipid Synthesis Pathways

**Figure 4:**
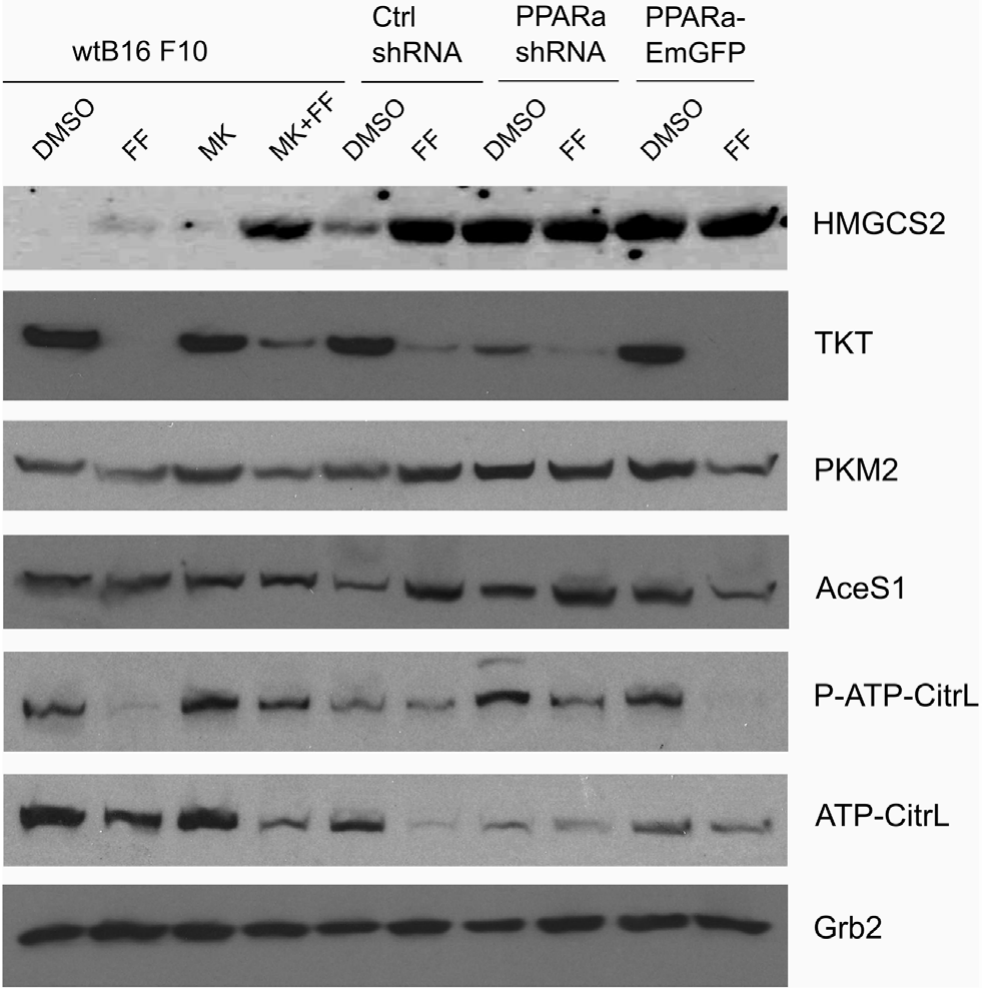
**HMGCS2 expression is inversely correlated with the level of enzymes involved in pentose phosphate pathway, transketolase (TKT) and the glycolytic enzyme pyruvate kinase PKM2, as well as lipogenic enzymes acetyl-CoA synthetase (AceS1) and ATP-citrate lyase (total and phospho-Ser455)**. Fenofibrate reduces the ATP-citrate lyase phospho- and total protein levels. Grb2 detection is a loading control. The relative changes (comparing to the signal from the DMSO-treated wt B16 F10 cells) of the protein band intensities in each condition (Table 1).

Recently published results from experiments on mice treated with fenofibrate revealed the complex transcriptional response in hepatocytes, which included: the activation of canonical PPARa regulated processes, such as mitochondrial and peroxisomal fatty acid oxidation and ketogenesis; *de novo* fatty acid synthesis, elongation, and desaturation; as well as an increase in pentose phosphate pathway (PPP) flux at the expense of suppressed glycolysis (51). Although the PPP is indispensable in all normal cells, including hepatocytes, it has recently gained recognition as being far more important to transformed cells. It not only provides NADPH for macromolecule biosyntheses and antioxidant protection but also provides monosaccharide intermediates for nucleotide synthesis, both of which are the major requirements to carry on proliferation (52, 53). Therefore, we decided to inspect the level of some of the critical enzymes involved in the PPP and lipid biosynthesis in the B16 F10 sub-lines with different PPARa expression. We noticed an intriguing, inverse correlation between HMGCS2 expression, which is upregulated by fenofibrate, and TKT – the key PPP enzyme – linking the oxidative and non-oxidative branches of this pathway. Figure 4 shows that the wt B16 F10 and Ctrl shRNA cells expressed higher levels of HMGCS2 after fenofibrate treatment. At the same time, in all cell lines fenofibrate had significantly downregulated the TKT level. Consequently, lower TKT levels were accompanied by lower levels of acetyl-CoA synthetase 1 (AceS1), phospho- and total ACL, and pyruvate kinase isoform M2 (PKM2) (Figure 4; Table 1). AceS1 is a cytoplasmic enzyme that catalyzes the conversion of acetate into acetyl-CoA, which is necessary for lipid synthesis, while ACL facilitates the transport of acetyl-CoA from the mitochondria to the cytoplasm to provide substrates for lipid synthesis and gluconeogenesis. ACL phosphorylation at Ser 455 (by PKA and Akt) enhances its enzymatic activity (54–56). PKM2 is an isoform of suboptimal affinity for the substrate, so the reaction rate is not as high as with other pyruvate kinase isoforms. PKM2 is highly expressed in embryonic and cancer cells, where it is believed to help them to spare glycolytic intermediates for the synthesis of macromolecules, such as lipids. Taken together, it appears that intensive ketogenesis, induced by fenofibrate, is associated with a downregulation of fatty acid biosynthetic pathways.

**Table 1:** **Densitometry of the immunoblotting results from Figure 4: protein band densitometry (band intensity normalized to Grb2 signal and wt B16 F10 DMSO, mean±SD)**.

Cell line	Treatment	Transketolase (TKT) (*n*=3)	Pyruvate kinase (PKM2) (*n*=5)	Acetyl-CoA synthetase (AceS1) (*n*=3)	Phospho-ATP-citrate lyase (*n*=3)	ATP-citrate lyase (*n*=3)
wt B16 F10	DMSO	1.00	1.00	1.00	1.00	1.00
	FF 50 μM	0.20 ± 0.078	0.74 ± 0.286	0.90 ± 0.119	0.54 ± 0.208	0.76 ± 0.296
	MK886 10 μM	0.93 ± 0.361	1.06 ± 0.412	0.94 ± 0.131	0.98 ± 0.381	0,99 ± 0.383
	FF + MK886	0.16 ± 0.063	0.78 ± 0.302	0.88 ± 0.226	0.84 ± 0.327	0.73 ± 0.082
Ctrl shRNA	DMSO	1.18 ± 0.420	0.96 ± 0.371	0.79 ± 0.458	0.62 ± 0.239	1.00 ± 0.390
	FF 50 μM	0.08 ± 0.033	0.69 ± 0.267	1.01 ± 0.102	0.55 ± 0.214	0.55 ± 0.212
PPARa shRNA	DMSO	0.43 ± 0.167	0.48 ± 0.184	0.92 ± 0.049	0.76 ± 0.293	0.42 ± 0.164
	FF 50 μM	0.18 ± 0.068	0.62 ± 0.240	1.01 ± 0.344	0.65 ± 0.252	0.42 ± 0.164
PPARa-EmGFP	DMSO	0.88 ± 0.343	0.45 ± 0.175	0.93 ± 0.086	0.72 ± 0.281	0.51 ± 0.197
	FF 50 μM	0.22 ± 0.084	0.38 ± 0.148	0.59 ± 0.113	0.34 ± 0.134	0.52 ± 0.201

### Ketogenesis Is Accompanied by an Arrest in Proliferation

**Figure 5:**
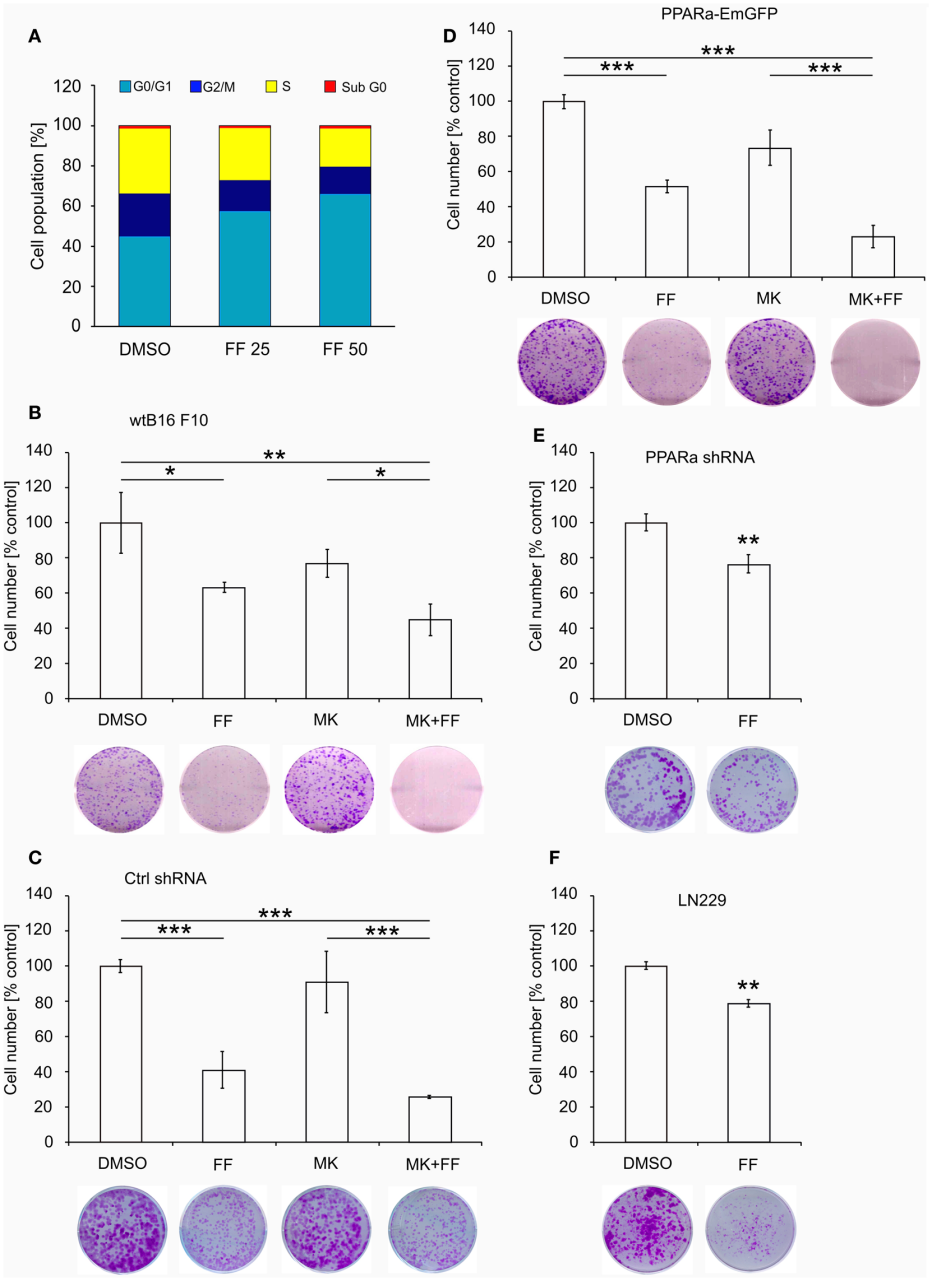
**Fenofibrate induces cell cycle arrest in the G0/G1 phase in a dose-dependent manner in B16 F10 cells (A)**. The graphs **(B–F)** present the changes in cell numbers in melanoma (wt B16 F10, Ctrl shRNA, PPARa shRNA, and PPARa-EmGFP) and glioblastoma (LN229) cells after the 48 h incubation with fenofibrate (FF, 50 μM), the PPARa inhibitor MK886 (MK, 10 μM), or both. DMSO was used as a control. The photos under each graph show the representative dishes from the clonogenic assays, stained with crystal violet.

**Figure 6:**
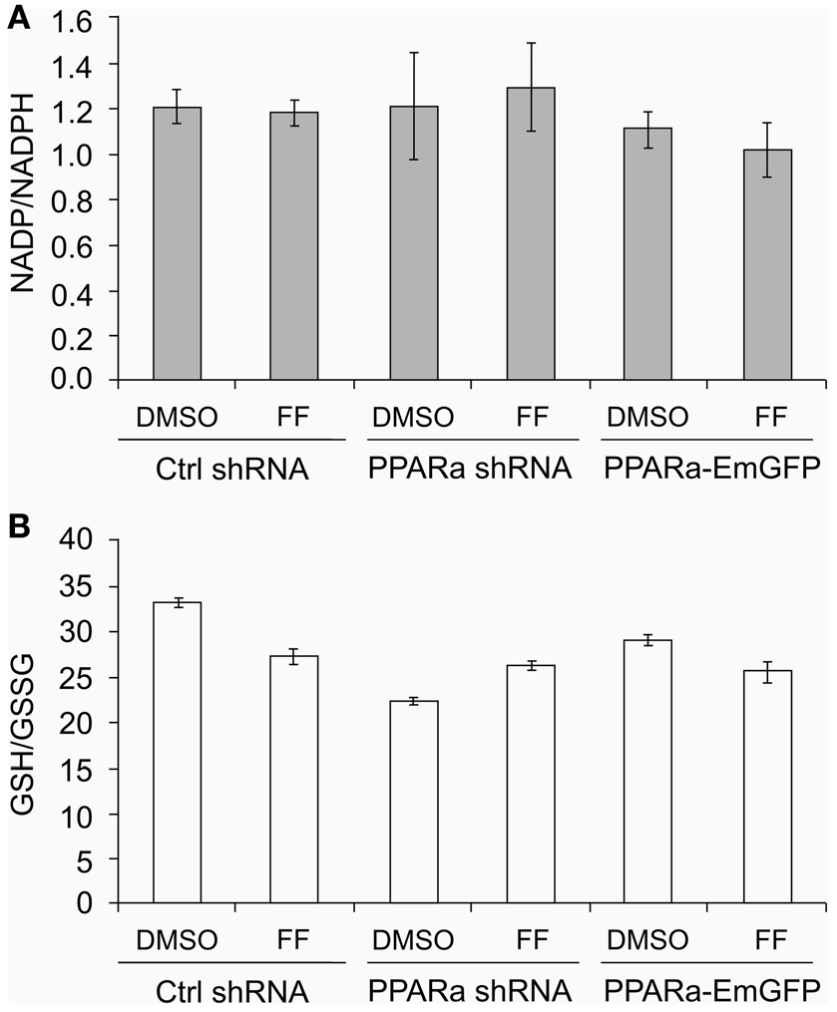
**Fenofibrate does not alter the NADP to NADPH (A) nor the GSH to GSSG (B) ratios in B16 F10 cells, regardless of PPARa level (PPARa control level – Ctrl shRNA, PPRAa knock down – PPARa shRNA, PPARa overexpression – PPARa-EmGFP)**.

Similarly to our previously reported results (49, 57), fenofibrate induced growth arrest and blocked proliferation of both B16 F10 and LN229 cells. Fenofibrate increased the percentage of quiescent cells (G0/G1 phase) while concomitantly reducing the fraction of cells in S and G2/M phases (Figure 5A). Moreover, FF treatment also blocked colony formation (Figures 5B–F) in a PPARa-independent manner, since the PPARa inhibitor MK886 was not able to reverse this effect (Figures 5B–D). Nucleotide and lipid biosynthesis are indispensable for sustaining proliferation and both require a supply of NADPH. Downregulation of TKT may negatively influence the course of PPP and consequently NADPH generation. We suspected that such a decrease in the NADPH/NADP ratio might be responsible for the slowdown in macromolecule synthesis and impaired antioxidant protection, which largely relies on both GSH levels and NADPH-dependent glutathione peroxidase activity. A shortage of macromolecules for membrane and DNA synthesis as well as reduced protection from oxidative stress are both sufficient to induce growth arrest; thus, we decided to estimate changes in NADP/NADPH and GSH/GSSG ratios in B16 F10 cells treated with fenofibrate. We did not detect any statistically significant changes in the NADP/NADPH ratio (Figure 6A). In the case of glutathione, fenofibrate indeed moderately decreased the GHS/GSSG ratio in cells with normal PPARa levels (ctrl shRNA); however, the changes were insignificant (Figure 6B). In cells with PPARa depletion or overexpression, the effect of fenofibrate was hardly visible. Altogether, this suggests that the oxidative branch of the PPP, which directly produces NADPH or NADPH-generating malic enzyme, is active enough to maintain NADPH/NADP homeostasis, despite the fenofibrate-driven reduction of TKT levels. The cells also cope quite well and maintain a fairly constant GSH/GSSG ratio, which suggests that an oxidative stress is not the reason for the inhibition of proliferation.

## Discussion

Production and release of ketone bodies has never been observed in any transformed cells. It was believed that not only particular types of normal cells and primarily hepatocytes but also kidney and intestinal epithelial cells, as well as astrocytes, were able to carry out ketogenesis in particular physiological conditions, such as prolonged fasting, a KD, or a calorie restricted ketogenic diet (CRKD).

In this study, we show that cancer cells of neuroepithelial origin, namely melanoma and glioblastoma, can abundantly produce bHB when treated with fenofibrate, a potent PPARa pharmacological agonist and a hypolipidemic drug. Fenofibrate promotes ketogenesis not only in the malignant cells but also in neurospheres composed of neurons and glia (Figure 2D). Although it is generally established that PPARa is the main transcription factor responsible for the induction of fatty acid catabolism and subsequent ketogenesis, in our experimental setting, the fenofibrate-induced bHB production seemed to be a PPARa-independent phenomenon. Neither the knock down of PPARa protein level nor its overexpression affected the ability of cells to produce and release bHB. Similarily, the effect of fenofibrate was not reversed when the PPARa transcriptional activity was blocked by MK886. Ketogenesis activated by fenofibrate started after only 6 h of treatment as manifested by HMGCS2 upregulation, but the cells needed about 48 h to fully adapt to this process (Figure 3B). Interestingly, both fenofibrate and the PPARa inhibitor MK886 also upregulated the levels of cytoplasmic HMGCS1, an enzyme involved in cholesterol synthesis.

The capacity of cancer cells to produce ketone bodies described here is quite different from previous reports, including series of papers (58–60) by the Lisanti group and coworkers regarding the possibility of ketogenesis in stromal fibroblasts surrounding breast cancer lesions. In those papers, the authors showed that cancer cells actively contributed to the stromal fibroblasts’ metabolic reprograming and took advantage of the subsequent ketone body consumption for energy generation. This is a special property of epithelia-derived tumors, such as breast carcinomas, since other cancer types do not express the proper enzymatic machinery for ketone body utilization as a source of energy (25, 28, 34). More often, cancers such as neuroblastomas or astrocytomas do absorb ketone bodies, but they use them for lipid synthesis rather than energy production (61–63). We cannot exclude the possibility that the cells in our study reabsorb and utilize some bHB, but neither B16 F10 melanoma nor LN229 glioblastoma employ it to obtain a growth advantage.

Recently, it has emerged that ketone bodies play much more complex roles than merely providing energetic substrates or metabolic intermediates, as they can also act as signaling molecules (64). On one hand, absorption and utilization of bHB decreases the intracellular NAD to NADH ratio and therefore affects the activity of sirtuins, but on the other hand, bHB is an inhibitor of class I histone deacetylases (HDAC 1, 2, 3, 8) and class IIa histone deacetylases (HDAC 4, 5, 7, 9) that bind zinc atoms at the active site (24, 64). The suppression of HDAC activity leads to various epigenetic modulations associated with global histone hyperacetylation. In this way, bHB exerts pleiotropic effects. For example, it induces the stress response gene FOXO3A, which is a tumor suppressor gene responsible for cell cycle arrest, reactive oxygen species detoxification, and apoptosis in various stressogenic conditions (65, 66). Indeed, in our previous studies, we observed the accumulation of FOXO3A in the nucleus and enhanced transcriptional activity of this protein in LN229 glioblastoma cells treated with fenofibrate. We also noted that FOXO3A was responsible for triggering apoptosis following prolonged exposure to fenofibrate (49). In light of our results, it is possible that bHB released by LN229 cells treated with fenofibrate can activate an autocrine loop and drive FOXO3A translocation and activation.

Melanoma and glioblastoma cells rarely derive a significant amount of energy from fatty acid beta-oxidation, although some advanced melanoma cells have been shown to upregulate enzymes involved in fatty acid catabolism and OXPHOS (67). In this study, however, we did not detect changes in the protein levels of enzymes involved in mitochondrial and peroxisomal fatty acid oxidation, namely acyl-CoA dehydrogenase and acyl-CoA oxidase, respectively (our unpublished data). Moreover, fenofibrate severely reduced mitochondrial oxygen consumption (Figure 3D), which suggests possibility that the peroxisomal beta-oxidation was actually responsible for the generation of acetyl-CoA needed for ketone bodies synthesis. The non-mitochondrial oxygen consumption level (the level that was retained after rotenone injection, Figure 3D) was not affected by fenofibrate treatment. This would suggest that fenofibrate forces the cancer cells to oxidize fatty acids in peroxisomes, which does not result in ATP production (ATP is not generated in peroxisomes). This may lead to the severe energetic stress that we have observed previously (48, 49). This energetic stress certainly contributes to the slowdown of proliferation and cell growth arrest as demonstrated by cell cycle analysis and colony formation assays (Figure 5).

Puzzled by the cellular response to fenofibrate, we tried to determine which other metabolic pathways are altered following treatment. We found that there was a strong downregulation of TKT, the main enzyme of the non-oxidative branch of the PPP. The non-oxidative branch of the PPP is responsible for monosaccharide backbone conversions leading to the synthesis of ribose precursors, but is not a source of NADPH. Indeed, the NADP to NADPH ratio in the cells treated with fenofibrate remained unaffected (Figure 6A). Importantly, the non-oxidative PPP branch is indispensable for ribose synthesis in tumor cells, where it provides between 70 and 85% of ribose stocks (52, 68). Therefore, the downregulation of TKT by fenofibrate and subsequent limitation of ribose flux may be another factor contributing to the cell growth arrest seen following fenofibrate treatment. The PPP is active in most tissues and almost all cells need sugar phosphate intermediates to produce not only nucleotides for nucleic acid synthesis but also crucial nucleotide derivatives, such as NAD(P), FAD, ATP, and CoA-SH.

Melanoma cells are able to sustain NADPH levels and maintain a fairly constant NADP/NADPH ratio, most likely through other NADPH reducing reactions either by NADP-dependent malic enzymes or nicotinamide nucleotide transhydrogenase (NNT). Malic enzyme catalyzes the oxidative decarboxylation of malate to pyruvate with a concomitant reduction of NADP to NADPH. Malic enzyme’s isoforms ME2 and ME3 reside in mitochondria whereas ME1 is cytosolic, and all of them are frequently overexpressed in tumor tissues of diverse origin (69–73). Recently, a reciprocally opposite regulation between p53 and ME1 and ME2 has been reported, showing that p53 knock out increases malic enzymes expression and that this gives the tumor cells a growth advantage, facilitating glutaminolytic flux and lipogenesis (74). Transhydrogenase NNT is a mitochondrial inner membrane protein that functions as a proton pump and simultaneously transfers protons and electrons from one type of reducing equivalent (NADH) to another (NADPH) (75). This enzyme has also garnered interest in the context of cancer metabolism, because it replenishes mitochondrial NADPH stocks required for isocitrate dehydrogenase-driven reductive carboxylation of a-ketoglutarate to isocitrate, which is employed by cancer cells to enhance lipogenesis (76).

In conclusion, it is quite surprising that fenofibrate is able to reprogram melanoma and glioblastoma metabolic pathways in such a way that they suffer from an energy deficit but are still forced to produce ketone bodies. This finding is important because, as far as we are aware, ketogenesis in cancer cells has not been described before and the underlying mechanism still waits to be revealed. The ketone bodies released by glioblastoma cells could serve not only as a fuel but also as a cytoprotective signaling molecule for neurons in the microenvironment surrounding the tumor tissue. The inability of neuroectodermal cancers to metabolize ketone bodies for their own benefit was long ago put forth as the rationale for the implementation of a KD as a therapeutic option for brain tumors, and it was later supported with experimental and clinical evidence (41, 77–80). Our results seem to further support this notion, as well as suggest some pharmacological agents, such as fenofibrate, as a supplement for such dietary therapeutic regimens.

## Author Contributions

MG: involved in research design, preparation of the manuscript, and experimental work including cell culture, clonogenic growth and immunoblotting; AW: design and execution of metabolic profiles using Extracellular Flux analyzer; AA and EW-T: assisted Dr. Grabacka in all experimental aspects of the work; PB: cloning strategies for of PPAR vectors; MD: evaluation of ketone bodies in cells following fenofibrate treatment; MP: imaging and immunocytofluorescence; KR: research design and editorial work on the manuscript.

## Conflict of Interest Statement

The authors declare that the research was conducted in the absence of any commercial or financial relationships that could be construed as a potential conflict of interest.
